# Infant with a hereditary blistering disorder: an interesting case in the NICU

**DOI:** 10.1093/omcr/omae041

**Published:** 2024-05-20

**Authors:** Rifkatou Tchignaha, Jessica Restivo, Christina Szialta, Oksana Nulman, Abhinav Parikh

**Affiliations:** Department of Pediatrics, New York Presbyterian Brooklyn Methodist Hospital, Brooklyn, New York, USA; Department of Rehabilitation Medicine, Occupational therapy, New York Presbyterian Brooklyn Methodist Hospital, Brooklyn, New York, USA; Department of Pediatric, Genetics, New York Presbyterian Brooklyn Methodist Hospital, Brooklyn, New York, USA; Department of Pediatrics, Research, New York Presbyterian Brooklyn Methodist Hospital, Brooklyn, New York, USA; Department of Pediatrics, Neonatology, New York Presbyterian Brooklyn Methodist Hospital, Brooklyn, New York, USA

## Abstract

This is a case of hereditary skin disorder in a full-term female newborn, with family history of epidermolysis bullosa (EB), who developed skin vesicles on the first day of life (DOL) without mucosal or ocular involvement. A multidisciplinary approach involving dermatology, wound care, and occupational therapy led to full recovery in our patient within six days of life. Special precautions were taken to prevent complications. Upon genetic testing, the patient was found to have a genetic variant of unknown significance (VUS). The goal of this case report is to give a detailed account of the patient’s course, provide management recommendations which could be applied to similar cases and settings in the newborn period.

## INTRODUCTION

Inherited blistering disorders, including Epidermolysis Bullosa (EB) are lifelong, rare genetic skin conditions affecting underlying structural proteins, with varying severity [1]. The burden on caregivers is significant enough to warrant a standard of care. This case presents an account of a hereditary blistering disorder with genetic variant of unknown significance (VUS), and provides management recommendations for future cases.

EB affects 8.2 per million live births in the United States [2]. According to the 2020 classification, the four types are: EB simplex, junctional EB, dystrophic EB, and Kindle EB or mixed [2] based on symptom severity. Newborns generally present with skin fragility, blistering, infection, dehydration, and failure to thrive [3].

Current guidelines recommend a multidisciplinary approach, with emphasis on infection control. A combination of topical antiseptics (chlorhexidine, benzalkonium chloride and silver sulfadiazine) and topical antibiotics (mupirocin, fusidic acid) have been used to prevent bacterial resistance [4, 5]. Warm baths and medications such as acetaminophen, opioids and anxiolytics have been used to alleviate pain; antihistamines and daily emollients to prevent pruritus, irritation and dryness. Breastfeeding is recommended for infants, but nutritional optimization with increased calories is recommended for growth and wound healing [3].

## CASE REPORT

37- 4/7 week female was born to a 31-year-old G2P1011 mother via vaginal delivery. Infant was appropriate for gestational age, and weighed 3.260 kg. APGAR scores were 8 and 9. Physical examination was unremarkable, with intact skin without any rash; infant was admitted to the nursery.

**Figure 1:**
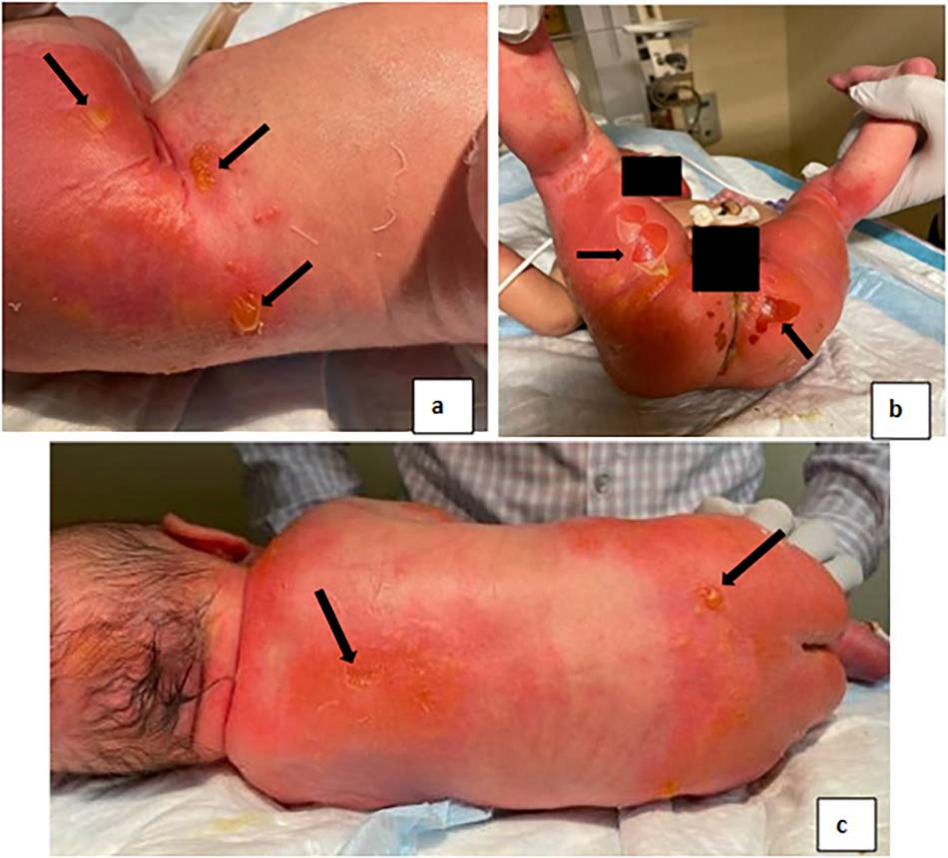
(**a**) Bullae, vesicles, with surrounding erythema on the left hip. (**b**) Unroofed bullae visible on the left, right thigh, and buttocks with surrounding erythema; evolving lesions on the right thigh. (**c**) Bullae on the upper and lower back with surrounding erythema.

On day of life (DOL) 1, shallow and flaccid bullae on the left hip (Fig. 1a) and erythema of the upper and lower back (Fig. 1c) were noted. There was no mucosal or ocular involvement, no nail dystrophy. Due to suspected EB given the family history, the infant was transferred to the Neonatal Intensive Care Unit (NICU) for further management.

Upon NICU admission, care was taken to avoid excessive friction to the skin, diaper elastic was avoided. The infant was on cardiopulmonary monitor with only the pulse oximeter on the foot, gauze dressing under the adhesive. Intravenous access was avoided, breastfeeding was encouraged with the help of lactation consultant, and intake and output were monitored. The basic metabolic profile was normal. The baby remained in the incubator with 60% humidity for thermoregulation.

### Treatment

Wound care, dermatology, and occupational therapy followed the patient. Bacitracin 500 units/gram and mupirocin 2% ointment were applied on sloughed areas twice daily. The entire skin was lathered with Aquaphor and petroleum jelly daily. Three layers of dressing were applied: a contact layer of silicone dressing on the intact and lanced bullae, a second layer with roll gauze, and netting/tubular dressing for securement. New bullae were lanced and drained in a sterile fashion, followed by application of bacitracin and mupirocin. As infection precaution, vesicles were not unroofed. Infant was placed on Z-flo mattress to limit pressure on affected areas. The skin was exposed to air, during cares four times per day, and foam boarders placed in between extremities to avoid friction. The patient received sweet-ease and acetaminophen 15 mg/kg per dose for pain during care and as needed.

**Figure 2:**
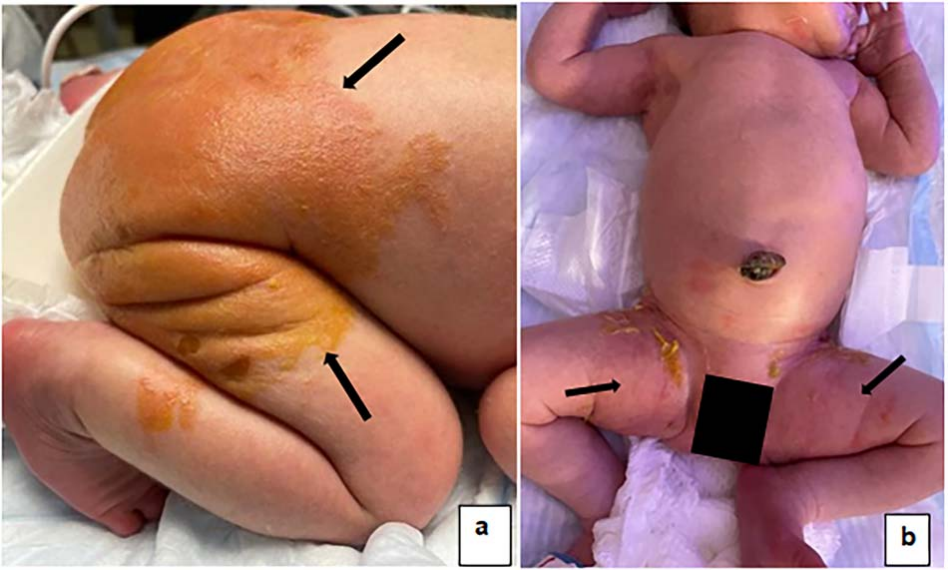
(**a**) Healing skin on the back, no new bullae or vesicles, no erythema. (**b**) Healing skin, no new bullae or vesicles.

On DOL 2, unroofed bullae and peeling were noted on the buttocks (Fig. 1b). Erythema was noted in the groin, extending onto the hips and flexor areas (Fig. 1b). On DOL 4, no new vesicles or bullae formed. The skin on the groin was minimally erythematous. On DOL 6, no new vesicles, erythema, or sloughing noted (Fig. 2a and b). Mother and family caregivers received extensive teaching throughout the NICU stay. She was discharged home with instructions to apply petroleum jelly several times daily to the diaper area, cover open lesions with petroleum jelly gauze, and if needed, follow with kerlix gauze.

The patient was seen outpatient by Pediatric Dermatology at one week of life; no new lesions noted. At three weeks old the patient had intact skin. Plan was made to start decolonization with dilute bleach baths at an older age; a process designed to protect skin from recurrent infections in the setting of a predisposing chronic skin condition [6].

### Family History

Maternal verbal history was significant for EB and severe allergies. Mother reported bullae since birth which improved with age. Maternal EB skin biopsy was reported negative, though no skin biopsy was performed on the patient. Similar skin lesions were also reported in maternal grandmother, great grandmother and great-great grandmother. Paternal family history was unremarkable. The patient is a product of a non-consanguineous union of Ashkenazi Jewish descent. The patient’s GeneDx Epidermolysis Bullosa Panel, which tests for 27 EB-associated genes revealed heterozygous VUS: *KRT1 c.1012T>C* p.(S338P) and PLEC c. 1012 T > C p.(S338P). The patient’s mother was later identified to have the KRT1 gene variant, associated with epidermolysis ichthyosis [7].

## DISCUSSION

Variants in the KRT1 gene are typically associated with autosomal dominant epidermolytic ichthyosis, with erythroderma, bullae, and large areas of denuded skin at birth. Skin fragility decreases with age, but patients develop severe hyperkeratosis with palmoplantar keratodermas over time. This variant has been previously reported with a mild phenotype: skin peeling at birth and mild hyperkeratosis developing later in life [7]. The mother reports skin findings consistent with mild KRT1/epidermolytic ichthyosis, a VUS by the American College of Medical Genetics (ACMG) classification; however, inheritance from a symptomatic family member and a previously published case report increase suspicion that this is a disease-causing variant [8]. Variants in the PLEC gene are associated with EBS [3], typically autosomal recessive, however in rare instances autosomal dominant variants have been reported in milder EBS. This variant is classified as a VUS by GeneDx, however it has conflicting reports of pathogenicity.

Multidisciplinary approach led to full recovery in our patient within six days. Our results are encouraging, but more studies are needed to establish standard of care for similar neonatal disorders. Electrolyte disturbances though absent in this case, should be monitored with larger affected BSA. Further investigation into both genetic variants may provide additional information into the clinical significance of the genetic findings, and lead to early detection and management options.
